# Enhancing Removal of Cr(VI), Pb^2+^, and Cu^2+^ from Aqueous Solutions Using Amino-Functionalized Cellulose Nanocrystal

**DOI:** 10.3390/molecules26237315

**Published:** 2021-12-02

**Authors:** Qinghua Xu, Xiaodi Huang, Lukuan Guo, Yu Wang, Liqiang Jin

**Affiliations:** 1 State Key Laboratory of Biobased Material and Green Papermaking, Qilu University of Technology (Shandong Academy of Sciences), Jinan 250353, China; lilihxd@163.com (X.H.); glk8362@163.com (L.G.); jcddjy121@126.com (Y.W.); 2 College of Light Industry Science and Engineering, Qilu University of Technology (Shandong Academy of Sciences), Jinan 250353, China

**Keywords:** cellulose nanocrystal, periodate oxidation, diethylenetriamine, adsorption, heavy metal ions

## Abstract

In this work, the amino-functionalized cellulose nanocrystal (ACNC) was prepared using a green route and applied as a biosorbent for adsorption of Cr(VI), Pb^2+^, and Cu^2+^ from aqueous solutions. CNC was firstly oxidized by sodium periodate to yield the dialdehyde nanocellulose (DACNC). Then, DACNC reacted with diethylenetriamine (DETA) to obtain amino-functionalized nanocellulose (ACNC) through a Schiff base reaction. The properties of DACNC and ACNC were characterized by using elemental analysis, Fourier transform infrared spectroscopy (FT-IR), Kaiser test, atomic force microscopy (AFM), X-ray diffraction (XRD), and zeta potential measurement. The presence of free amino groups was evidenced by the FT-IR results and Kaiser test. ACNCs exhibited an amphoteric nature with isoelectric points between pH 8 and 9. After the chemical modification, the cellulose I polymorph of nanocellulose remained, while the crystallinity decreased. The adsorption behavior of ACNC was investigated for the removal of Cr(VI), Pb^2+^, and Cu^2+^ in aqueous solutions. The maximum adsorption capacities were obtained at pH 2 for Cr(VI) and pH 6 for Cu^2+^ and Pb^2+^, respectively. The adsorption all followed pseudo second-order kinetics and Sips adsorption isotherms. The estimated adsorption capacities for Cr(VI), Pb^2+^, and Cu^2+^ were 70.503, 54.115, and 49.600 mg/g, respectively.

## 1. Introduction

Heavy metal ions are usually toxic and have a negative influence on the environment and human health, such as Cu^2+^, Pb^2+^, Cr(VI), Hg^2+^, etc. [1]. They can be removed from effluents by adsorption, coagulation and flocculation, ion exchange, chemical redox, and reverse osmosis. Due to the flexible operation, low energy consumption, low residue, and adsorbent reusability, adsorption has been considered to be more economical and efficient [2,3,4], especially for low-concentration (<100 mg/L) heavy metal ion wastewater. The selection of adsorbents is crucial for an adsorption process. Conventional adsorbents have limited surface area and adsorption sites; thus, their adsorption rate and capacity are usually limited. Nanomaterials with a large surface area and more active sites have drawn more and more attention and are expected to be more efficient in metal removal [5,6,7]. 

Cellulose is the most abundant biomass resource on Earth. It is considered as a candidate for environmentally friendly biobased materials to replace synthetic polymers from petroleum chemicals. Nanocellulose, which could be extracted from lignocellulosic materials, has drawn increasing attention in wastewater treatment as adsorbents due to its large specific surface area, high Young’s modulus, and rich surface groups [6,7,8,9]. Liu et al. [10] prepared cellulose nanocrystals (CNCs), cellulose nanofibers (CNFs), and chitin nanocrystals (ChNCs) from bioresidues and compared their adsorption performance on silver ions from contaminated water. Their adsorption behaviors were dependent on pH, and CNC gave the highest adsorption capacity. Nanocellulose fibers, prepared from rice straw, demonstrated a considerable adsorption efficiency of Cd(II), Pb(II), and Ni(II) ions [11]. 

The heavy metal ions in the effluent are complicated and vary depending on the origin of the industry. Nanocellulose without modification can only be used to adsorb positively charged ions; however, some heavy metals occur as anions, such as Cr(VI), Mo(VI), Au(III), Se(V), etc. It is desirable that the adsorbents can efficiently remove both positively and negatively charged ions. Chemical modification of nanocellulose is a promising way to increase the adsorption ability and adaptability to variety of contaminants. Hokkanen et al. [12] modified microfibrillated cellulose (MFC) using carbonated hydroxyapatite (CHA), which exhibited a higher adsorption capacity for Ni^2+^, Cd^2+^, PO_4_^3−^, and NO_3_^−^ from aqueous solutions than those of unmodified MFC. Kyzas et al. [13] prepared chitosan-based adsorbents by grafting with *N*-(2-carboxybenzyl) firstly and then cross-linking with gultaraldehyde. It could effectively adsorb not only positively charged ions (Cu^2+^ and Ni^2+^), but also negatively charged ions (Cr(VI), As(V)). The primary amino groups and hydroxyl groups on chitosan molecular impart an adsorptive ability for different charged ions. After amino functionalization, nanocellulose also carries amino and hydroxyl groups, and is expected to exhibit similar adsorbing behavior with chitosan. Recently, amino-modified nanocellulose was proved to be a good candidate adsorbent towards heavy metal ions. Aminopropyltriethoxysilane (APS)-modified microfibrillated cellulose (MFC) was very effective at removing Ni(II), Cu(II), and Cd(II) from contaminated water [14]. Taleb et al. found that the adsorption performance of NC for arsenic was improved after amino-terminal functionalization [15]. Silva et al. prepared adsorption films with amino and carboxyl groups using CNFs and lysozyme nanofibers to adsorb Hg(II) from spring waters [16]. Xiong et al. developed an adsorbent using natural corn stalk (CS). The CS was firstly treated by sulfuric acid and then modified by ammonia (NH_3_)-thiosemicarbazide (TSC)-glutaraldehyde (TSC-NH_3_-OCS) [17]. The obtained adsorbent could selectively adsorb AgCl^1−i^ from the Ag(I)-Cu(II)-Ni(II) solution; however, toxic glutaraldehyde was used in the functionalization. Chai et al. [18] prepared a pH-sensitive nanocellulose adsorbent modified with polyethyleneimine (PEI) and glutaraldehyde, which was efficient in the removal of As(V) at pH 3. Glutaraldehyde was used as a crosslinker between TEMPO-oxidized nanocellulose and PEI. Aminated nanocellulose was prepared by epichlorohydrin-mediated amination and applied as an adsorbent for the removal of boric acid from aqueous solutions [19]. The maximum adsorption capacity was achieved at pH 7 by complexing with amines or hydroxyl on the adsorbent. 

In this work, we proposed a simple route for the amino functionalization of cellulose nanocrystal (CNC). CNC was firstly oxidized using sodium periodate to obtain the dialdehyde nanocellulose (DACNC), and then reacted with diethylenetriamine (DETA) to obtain amino-modofied nanocellulose (ACNC) through a Schiff base reaction between DETA and DACNC. Free amino groups were covalently bonded onto nanocellulose without using any toxic crosslinker, such as glutaraldehyde. The properties of ACNC were characterized by using elemental analysis, Fourier transform infrared spectroscopy (FT-IR), Kaiser test, atomic force microscopy (AFM), X-ray diffraction (XRD), and zeta potential measurement. ACNC was used as an adsorbent to adsorb Cu^2+^, Pb^2+^, and Cr (VI) from aqueous solutions, and its adsorption behaviors were investigated.

## 2. Results and Discussion

### 2.1. Preparation and Characterization of ACNC

**Figure 1:**
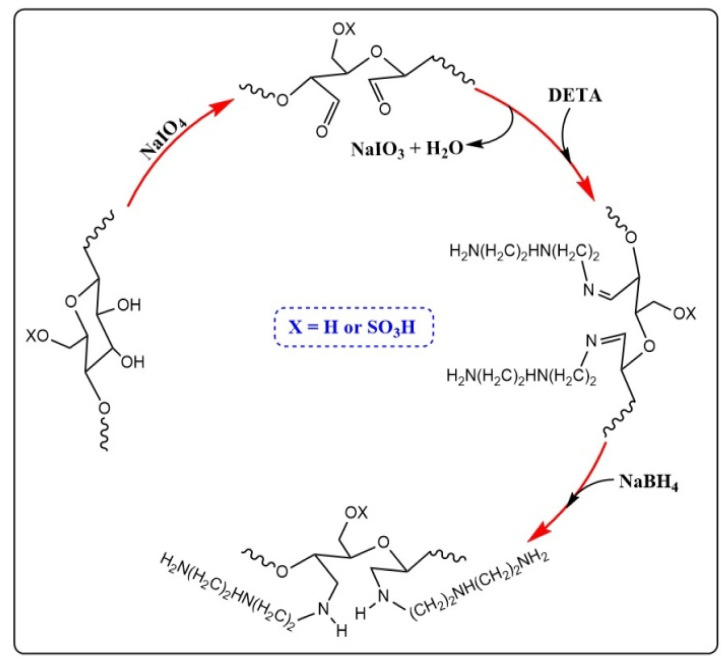
Schematic pathway for periodate oxidation and amino functionalization of CNC.

Figure 1 shows the schematic pathway for the oxidation and amino functionalization of CNC. CNC was firstly oxidized by sodium periodate to obtain dialdehyde nanocellulose (DACNC) with aldehyde groups at C-2/C-3 by ring opening. The aldehyde group content was determined to be 8.81 mmol/g. Then, the aldehyde groups on DACNC reacted with the primary amino groups on DETA through a Schiff-base reaction. Finally, ACNC with free amino groups was obtained by a reductive amination treatment using NaBH_4_.

**Figure 2:**
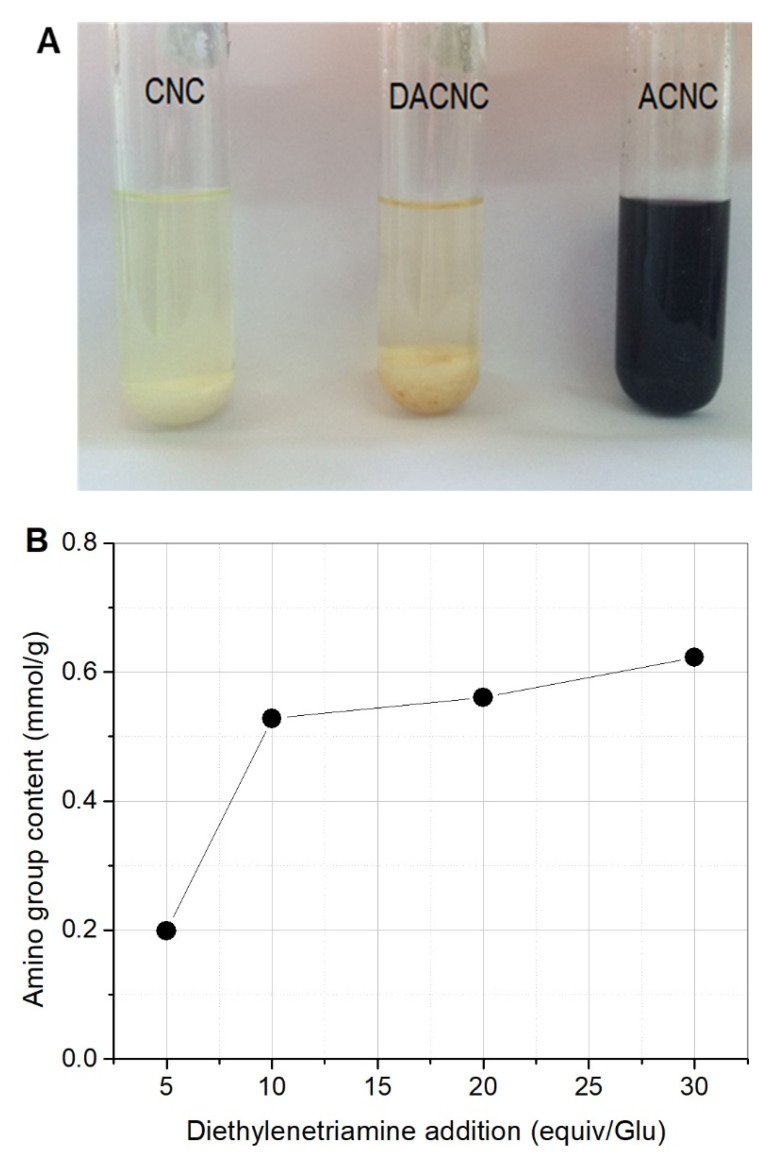
(**A**) Kaiser tests for CNC, DACNC, and ACNC; (**B**) Relationship between DETA and the amino group content of ACNC.

Kaiser test is a sensitive detection method for free amino groups. When there is no free amino group, the solution is yellow or colorless. The solution will turn blue when there is a free amino group. Kaiser tests were carried out for CNC, DACNC, and ACNC, and the results are shown in Figure 2A. The dark blue color of ACNC indicated the existence of the free amines. The primary amino group content of ACNCs was determined by conductometric titration, and the relationship between DETA addition and the amino group content of the obtained ACNC is shown in Figure 2B. The more DETA addition, the higher the amino group content of ACNC.

The contents of C, H, N, and S in CNC, DACNC, and ACNC samples were measured by using an elemental analyzer, and the results are listed in Table 1. It was found that CNC and DACNC did not contain any nitrogen, while nitrogen presented in ACNC obtained by DETA functionalization, suggesting the successful grafting of DETA onto nanocellulose. With the increase of DETA dosage, the content of nitrogen in the ACNC increased. A small amount of S was found in all the tested samples, which resulted from the H_2_SO_4_ hydrolysis of raw materials to produce CNC.

**Figure 3:**
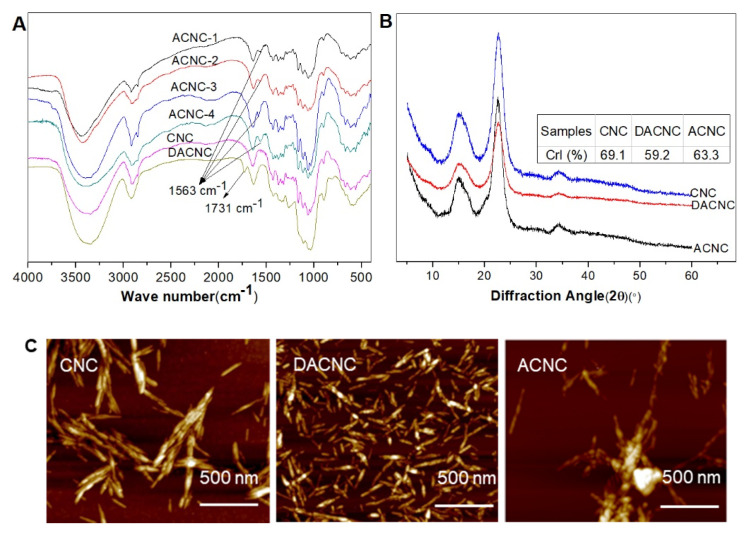
(**A**) FT-IR spectra, (**B**) X-ray diffraction patterns, (**C**) AFM images of CNC, DACNC, and ACNC.

The FT-IR spectra of CNC, DACNC, and ACNCs are illustrated in Figure 3A. Compared with the spectra of CNC, a new band appeared at 1731 cm^−1^ in the spectra of DACNCs, which was assigned to the C=O stretching vibration of the aldehyde groups. More DETA addition in the modification resulted in a higher intensity of this band. The presence of this new band indicated the successful oxidation of CNC to DACNC. However, in the spectra of ACNC, the band at 1731 cm^−1^ disappeared, and a new adsorption band at 1563 cm^−1^, which was assigned to N-H bending vibration, presented [20]. These results confirmed the successful reaction of DACNC with DETA. There are two characteristic N-H bands for free amines in the region of 3300 cm^−1^. They were not observed in the spectra of ACNC, while a broad band in this region corresponded to O-H stretching presented. They were probably overlapped with this O-H bond, as previously reported by Jin et al. [21]. 

The wide-angle X-ray diffraction patterns of the CNC, DACNC, and ACNC samples (Figure 3B) show characteristic peaks of cellulose I at 2θ = 14–18°, 22.5°, and 34.5° [22]. After the oxidation and amino functionalization, the cellulose I structure was retained, while the crystallinity index decreased as demonstrated in the inset table. During the periodate oxidation, some of the glucopyranose rings opened, and the ordered structure subsequently destroyed, resulting in the decrease of the crystallinity [23,24]. After the reaction with DETA, CrI increased a little from 59.2% to 63.3%, which may be due to degradation of some open structures. The decreased yield of amino nanocellulose (data not shown in this paper) could confirm this. 

Figure 3C shows the AFM images of the CNC, DACNC, and ACNC-4 samples. It could be seen that the surface morphology of nanocellulose did not change much before and after chemical modification, which retained the shape of nanowhiskers. The diameter of DACNC was slightly smaller than that of CNC. This agreed well with the result of Sun et al. [25], who also found a decreased diameter of periodate oxidized fibers. After DETA-functionalization, some small flocculations were observed, which is probably a result of the crosslinkage between CNC through DETA. It could be seen that the flocculation was very loose, and the adsorption behavior was not influenced.

**Figure 4:**
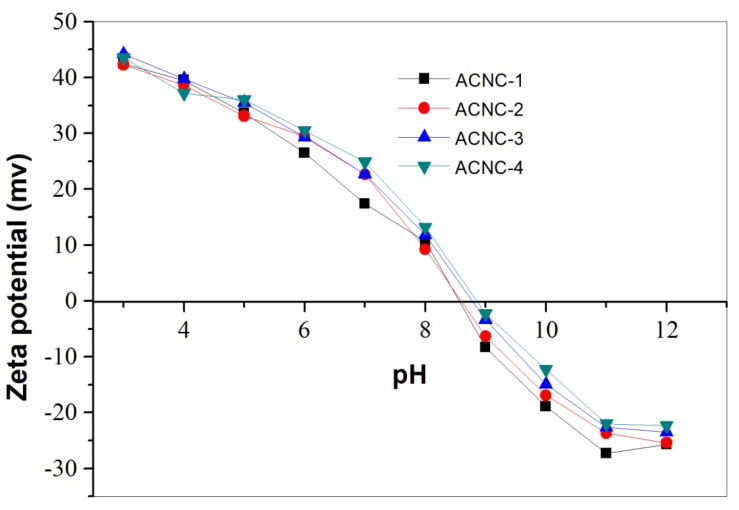
Zeta potential of ACNC samples at varied pH.

Figure 4 demonstrates the zeta potential of ACNC samples at different pH values. It was found that the zeta potential was significantly influenced by the pH. ACNC exhibited an amphoteric nature, and the isoelectric point (pH_pzc_) were between pH of 8 and 9, respectively. At acidic and neutral conditions, the free amine groups of ACNC protonated and thus gave a positive zeta potential. In the alkaline region, the zeta potential decreased gradually and became negative due to the deprotonation of amino groups and dissociation of sulfate groups from CNC. More DETA addition led to a higher isoelectric point, which is probably due to the higher content of primary amino groups on the obtained ACNC. Hokkanen et al. prepared aminopropyltriethoxysilane (APS)-modified microfibrillated cellulose (MFC) with free amino groups [14], which also show an amphoteric property with an isoelectric point of 5.65. The pH_pzc_ of ACNC in this work shifted to higher pH, which was in agreement with its higher amino group content.

### 2.2. Adsorption of Metal Ions

#### 2.2.1. pH Effect on Adsorption

**Figure 5:**
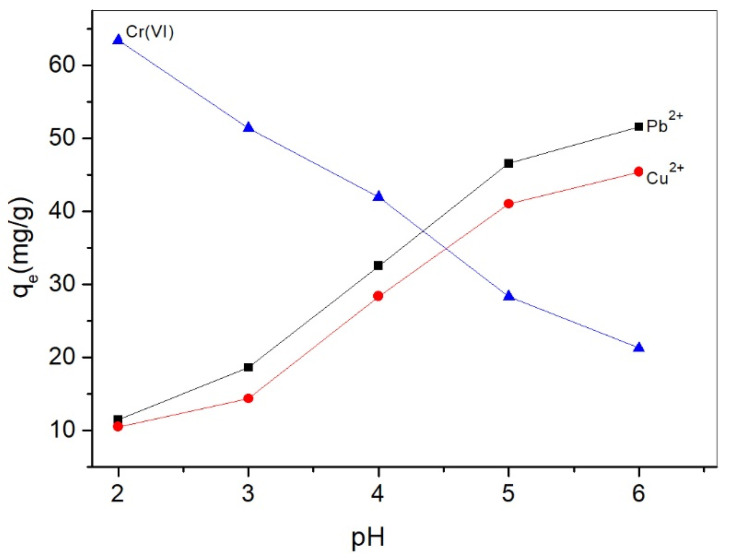
pH effect on the adsorption of Cr(VI), Cu^2+^, and Pb^2+^ onto ACNC (with a metal ion concentration of 100 mg/L and ACNC dosage of 0.5 g/L).

Figure 5 shows the pH influence on the Cr(VI), Cu^2+^, and Pb^2+^ adsorption on ACNC. The solution pH affects both the chemical species distribution of metal ions and the surface charge of the adsorbent, and subsequently influences the adsorption behaviors. In this study, the adsorption capacity at pH between 2 and 6 was measured to avoid the interference of metal precipitation above the pH range of 7.0–7.5 [26]. It was found that the adsorption capacities of Cr(VI), Cu^2+^, and Pb^2+^ on ACNC were greatly influenced by solution pH. For Cr(VI), ACNC exhibited the maximum adsorption capacity at pH 2, which agreed well with the report of Nakano et al. [27]. Cr(VI) is negatively charged at acid conditions. It exists mainly as HCrO_4_^−^ in aqueous solution with pH below 4.0, while CrO_4_^2−^ and Cr_2_O_7_^2−^ at pH above 4. At lower pH, ACNC carried more positive charge (as shown in Figure 4) and had more opportunities to adsorb Cr(VI) due to the electric attraction. The adsorption capacities for Pb^2+^ and Cu^2+^ increased with the increase of pH, and both reached the maximum at pH 6. In the pH between 2 and 6, Cu^2+^ and Pb^2+^ were the main species [28]. At lower pH, metal ions and ACNC were both positively charged, and the strong electrostatic repulsion between them led to a lower adsorption capacity. With the increase of the solution pH, more amino groups became neutral and the zeta potential of ACNC became lower, which reduced the electrostatic repulsion between the adsorbent and metal ions, and subsequently enhanced the adsorption capacities.

#### 2.2.2. Adsorption Kinetics

**Figure 6:**
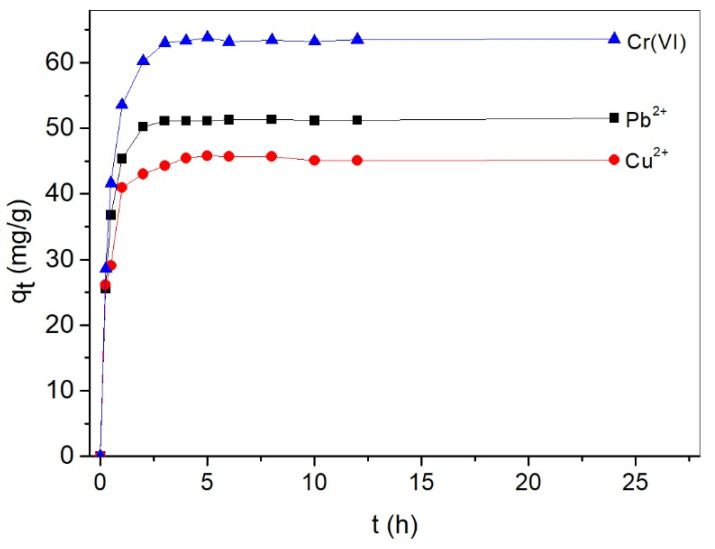
Influence of time on the adsorption capacities of Cr(VI), Cu^2+^, and Pb^2+^ on ACNC (with a metal ion concentration of 100 mg/L and ACNC dosage of 0.5 g/L).

The relationship between the adsorption capacities of Cu^2+^, Pb^2+^, and Cr(VI) and contact time is shown in Figure 6. For all three metal ions, the adsorption rate was very fast during the first hour. The adsorption capacities increased slowly after the fast uptake of one hour, and achieved equilibrium within 3 h. The adsorption followed the order of Cr(VI) > Pb^2+^ > Cu^2+^ at all time intervals. 

The adsorption kinetics of these three metal ions on ACNC were analyzed using pseudo first-order and pseudo second-order models. The linearized equations of the models are as follows:

(1) $$\mathrm{Pseudo}\;\mathrm{first}-\mathrm{order}:\;\log(q_{e}-q_{t})=\log q_{e}-k_{1}t/2.303$$

(2) $$\mathrm{Pseudo}\;\mathrm{second}-\mathrm{order}:\;{t/q}_{t}=1/\left(k_{2}q_{e}{}^{2}\right)+t/q_{e}$$

where q_e_ and q_t_ are the adsorption capacities per gram of ACNC at equilibrium and at time t, respectively, mg/g; and k_1_ and k_2_ are the rate constant for the pseudo first order and second order, respectively.

The kinetic parameters were calculated based on the above equations and the results are demonstrated in Table 2. The R^2^ values for the second-order model were all >0.99 for the three metal ions, while R^2^for the first-order model were much lower (0.660, 0.614, and 0.598 for Cr(VI), Pb^2+^ and Cu^2+^, respectively). q_e_ calculated using the second order model also matched well with the q_e_ obtained by the adsorption experiments. All these results suggested that the pseudo second-order model could better express the adsorption kinetics of Cu^2+^, Pb^2+^, and Cr(VI) on ACNC. Adsorption behavior that fits pseudo-second order well can often be explained by a diffusion-based mechanism [29]. 

#### 2.2.3. Adsorption Isotherms

The adsorption isotherm models of Langmuir, Freundlich, Sips, and Toth were used to calculate the adsorption parameters of Cr(VI), Cu^2+^, and Pb^2+^ onto ACNC. For the Langmuir isotherm model, it is assumed that the adsorbent is uniform, and a monolayer of adsorbate is adsorbed [30]. It could be expressed using the following equation:

(3) $$q_{e}=\frac{q_{m}K_{L}C_{e}}{1+K_{L}C_{e}}$$

where q_e_ and q_m_ are the equilibrium and maximum adsorption capacity, respectively, mg/g; C_e_ is the equilibrium concentration of the adsorbate, mg/L; and K_L_ is the Langmuir adsorption constant, L/g.

The Freundlich isotherm model describes that adsorption happens on heterogeneous surfaces with multiple adsorption layers [31]. It could be expressed by the equation below:

(4) $$q_{e}=K_{f}C_{e}{}^{1/n}$$

where q_e_ and C_e_ are the adsorption capacity and the adsorbate concentration at equilibrium; and K_f_ and 1/n are the two Freundlich constants. 

The Sips isotherm is a combination of the Langmuir and Freundlich models, which could be expressed as Equation (5):

(5) $$q_{e}=\frac{q_{s}{\left(K_{S}C_{e}\right)}^{m}}{1+{\left(K_{S}C_{e}\right)}^{m}}$$

where q_s_ is the specific adsorption capacity at saturation, mg/g; K_S_ is Sips isotherm constant, mL/mg; and m is the heterogeneity factor.

**Figure 7:**
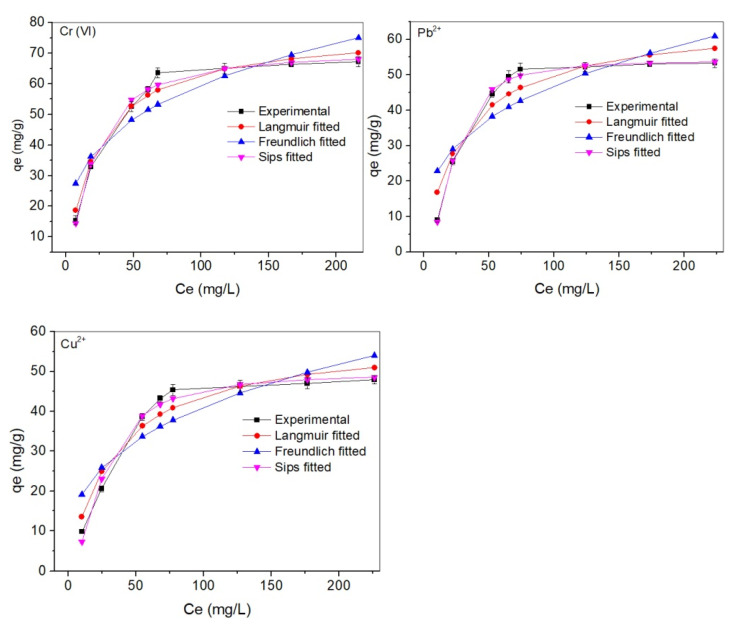
Langmuir, Freundlich, and Sips isotherm fitting for the adsorption of Cr(VI), Pb^2+^, and Cu^2+^on ACNC.

The Langmuir, Freundlich, and Sips isotherm fitting results for adsorption of Cr(VI), Cu^2+^, and Pb^2+^ on ACNC are shown in Figure 7 and the calculated parameters are demonstrated in Table 3. For all three metal ions, the Sips model gave the best description of the adsorption behavior with R^2^ > 0.98, while 0.759–0.970 for Langmuir and Freundlich models. The q_max_ values estimated by the Sips model (67.216, 53.258, and 47.913 mg/g for Cr(VI), Pb^2+^, and Cu^2+^, respectively) also agreed better with the q_e,exp_ than those estimated by the Langmuir and Freundlich models. The adsorption capacities for the three metal ions were in the order of Cr(VI)>Pb^2+^>Cu^2+^. A similar trend was observed in our previous report [32], in which we investigated the adsorption of Pb^2+^, Cu^2+^, and Cr(VI) using tannin-immobilized nanocellulose. K_L_ values, calculated by the Langmuir model, indicated the adsorption energies between the adsorbent and adsorbate. A higher K_L_ value means a faster adsorption. The K_L_ values in this work for the three metal ions were similar, which corresponded well with the results of the kinetic study.

Table 4 presents the comparison of the adsorption capacity for heavy metals between this work and some other functionalized nanocelluloses. Compared with pristine nanocellulose [8,9], functionalized ones gave higher adsorption capacity for Pb^2+^ and Cu^2+^. For Cr(VI), the adsorption capacity of ACNC was higher than amination CNCs with acrylamide and ethylenediamine [33] and lower than polycysteine-grafted CNFs [34]. The adsorption capacity of ACNC for Cu^2+^ was lower than carboxylic nanocellulose [35], almost the same as TEMPO-oxidized CNFs modified with PEI [36] and higher than TEMPO-modified CNFs [37]. Shen et al. prepared diethylenetriamine-bacterial cellulose (EABC) by pretreatment of BC with epichlorohydrin in alkaline conditions and then amination with diethylenetriamine [38]. Compared with that of EABC, the adsorption capacity of ACNC obtained in this work for Pb^2+^ was much higher, while it was lower for Cu^2+^.The adsorption capacity comparison suggested that ACNC is an efficient biosorbent for the removal of Cr(VI), Pb^2+^, and Cu^2+^.

#### 2.2.4. Reusability of ACNC

**Figure 8:**
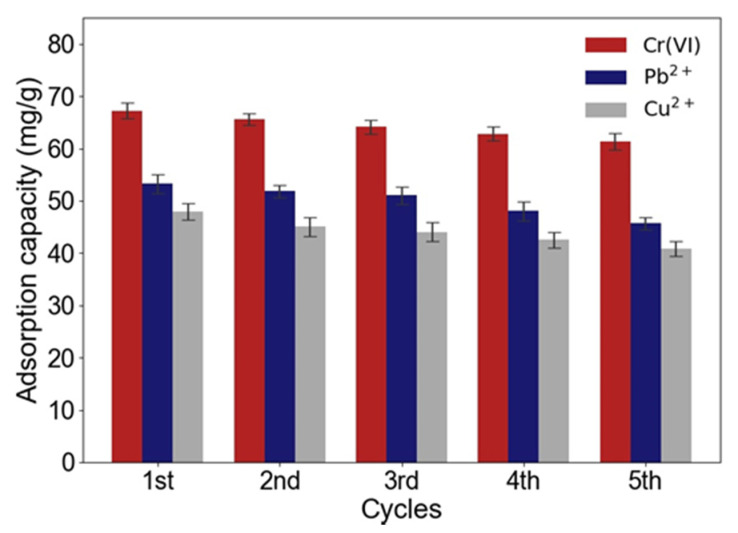
Adsorption capacity of ACNC before and after regeneration.

Adsorption-regeneration experiments were carried out for five cycles to evaluate the reusability of ACNC, and the results are shown in Figure 8. The adsorption capacity for Cr(VI), Pb^2+^, and Cu^2+^ all decreased slightly with the increasing number of cycles. However, only a 10–15% reduction was found after 5 cycles, suggesting a high reusability of ACNC. The covalent bondings between free amino groups and CNC were stable and durable during the adsorption and desorption process, leading to the high reusability of ACNC.

## 3. Materials and Methods

### 3.1. Materials

Cellulose nanocrystal (CNC) was provided by Tianjin Woodelfbio Cellulose Co., Ltd., Tianjin, China. Sodium periodate and DETA were purchased from Sigma-Aldrich Co. Ltd. Copper nitrate, Cu(NO_3_)_2_, lead nitrate, Pb(NO_3_)_2_, and potassium bichromate, K_2_Cr_2_O_7_, were purchased from Sinopharm Chemical Reagent Co. Ltd., China.

### 3.2. Preparation of DACNC

CNC was oxidized using sodium periodate to obtain DACNC based on the method reported by Jin et al. [21]. DACNC could react with hydroxylamine hydrochloride to obtain oxime and hydrochloric acid. The aldehyde group content could be determined by titration of the hydrochloric acid with sodium hydroxide, and calculated according to Equation (6):

(6) CHO(mmol/g)=C(V2−V1)/m

where C is the concentration of sodium hydroxide, mol/L; V_1_ and V_2_ is the volume of sodium hydroxide for titration of DACNC and CNC, respectively, mL; and m is the weight of the sample, g.

### 3.3. Amino Functionalization of DACNC with Diethylenetriamine (DETA)

In total, 200 mL of DACNC suspension (0.5wt%) were sonicated for 5 min, and then mixed with different amounts of DETA, 5 equiv/Glu, 10 equiv/Glu, 20 equiv/Glu, and 30 equiv/Glu, respectively. The reaction was carried out at 30 °C for 6 h. At the end of the reaction, 0.58 g of NaBH_4_ were added to reduce the obtained imine intermediate at room temperature for 3 h. The products were dialyzed (MWCO: 12,000–14,000) against deionized water to remove extra DETA and NaBH_4_. Four ACNC samples were obtained and named as ACNC-1, ACNC-2, ACNC-3, and ACNC-4, respectively, according to the amounts of DETA. The content of amino groups was measured by conductometric titration using sodium hydroxide according to the report of Filpponen et al. [39].

### 3.4. Characterization

#### 3.4.1. Elemental Analysis

The freeze-dried samples of CNC, DACNC, and ACNC were fully ground, and the percentages of C, H, N, and S were measured by using a Vario EL EL III elemental analyzer (Elementar Co. LTD, Langenselbold, Germany). The oxygen percentage could be calculated by deducing all the percentage of the above 4 elements.

#### 3.4.2. Zeta Potential Measurement

The zeta potential of nanocellulose samples before and after functionalization at varied pH was measured using a Zetasizer (Nano ZS90, Malvern Panalytical, UK).

#### 3.4.3. FT-IR

CNC, DACNC, and ACNC samples were ground into a powder and mixed with KBr to obtain KBr pellets. FT-IR spectra were obtained out on an FT-IR spectrometer (IRPrestige-21, Shimadzu Company, Japan) over a scan range of 400–4000 cm^−1^ at a resolution of 2 cm^−1^.

#### 3.4.4. X-ray Diffraction (XRD) Analysis

XRD patterns were performed on an X-ray Diffractometer equipped with a CuXa X-ray tube (D8, Bruker AXS, Germany). The crystallinity index (CrI) was calculated according to Equation (7) [40]:

(7) $$\mathrm{CrI}=\frac{I_{002}-I_{\mathrm{am}}}{I_{002}}$$

where I_002_ is the maximum intensity at 2θ between 22° and 23°, and I_am_ is the minimum intensity at 2θ between 18° and 19°.

#### 3.4.5. AFM Observation

The CNC, DACNC, and ACNC samples were diluted and deposited on a clean mica surface, and then air dried overnight. The surface morphology of these samples was observed using a Multimode 8 Nanoscope V System AFM (Bruker Corporation, Karlsruhe, Germany).

### 3.5. Adsorption of Metal Ions

ACNC-4 was used as the adsorbent in this work due to its highest amino group content. Firstly, Cu(NO_3_)_2_, Pb(NO_3_)_2_, and K_2_Cr_2_O_7_ were dissolved to prepare the stock solutions of Cu^2+^, Pb^2+^, and Cr (VI). The stock solutions were then diluted to the required concentrations, respectively. A certain amount of ACNC-4 was added into the Cu(NO_3_)_2_, Pb(NO_3_)_2_, and K_2_Cr_2_O_7_ solutions and stirred mildly. In total, 5 mL were withdrawn from the mixture every half or one hour, and then centrifuged at 4000 rpm for 5 min. The concentrations of Cu^2+^, Pb^2+^, and Cr(VI) were determined using an atomic absorption spectrometer (AAS6300, Shimadzu, Japan). The adsorption capacities were calculated based on Equation (8):

(8) $$q_{t}=\frac{\left(C_{0}-C_{t}\right)V}{m}$$

where q_t_ is the adsorbed amount after time t; C_0_ is the initial concentration of metal ions, mg/L; C_t_ is the concentration of metal ions after time t, mg/L; V is the volume of the solution, L; and m is the adsorbent weight, g.

### 3.6. Regeneration Study

The adsorbent was centrifuged after the adsorption and the metal ions-adsorbed ACNC was regenerated in 50 mL of 0.1 M nitric acid. The regenerated ACNC was then collected from the solution via centrifugation for reuse. This adsorption-regeneration cycle was repeated five times to test the reusability of ACNC.

### 3.7. Statistical Analysis

Each adsorption experiment was conducted in triplicate. The presented data was the mean value and standard deviations were also calculated.

## 4. Conclusions

Amino-functionalized nanocellulose (ACNC) with free primary amino groups was prepared from cellulose nanocrystal and diethylenetriamine using a green route without any toxic crosslinkers. The results of the FT-IR and Kaiser test evidenced the presence of primary amino groups on the obtained ACNC. ACNC samples exhibited an amphoteric nature with the isoelectric point (pH_pzc_) between pH of 8 and 9, respectively. The structure of cellulose I was maintained after chemical modification, while the crystallinity indices were reduced. ACNC could adsorb both positively charged Cu^2+^ and Pb^2+^, and negatively charged Cr(VI) by complexing amine with metal ions. The maximum adsorption capacity was obtained at pH 2 for Cr(VI), while pH 6 for Cu^2+^ and Pb^2+^, respectively. The adsorption of Cu^2+^, Pb^2+^, and Cr(VI) on ACNC fitted the pseudo second-order kinetics model and Langmuir isotherm model. ACNC was proved to be a good candidate as an efficient adsorbent to remove heavy metal ions from contaminated waters.

## Figures and Tables

**Table 1:** Elemental analysis of CNC, DACNC, and ACNC samples.

Samples	C	H	O	N	S
CNC	39.96	10.02	49.82	0	0.20
DACNC	39.75	9.27	50.73	0	0.25
ACNC-1	41.64	9.30	47.23	1.59	0.24
ACNC-2	41.24	9.66	47.10	1.71	0.29
ACNC-3	41.38	10.07	46.35	1.92	0.28
ACNC-4	41.64	10.40	45.63	2.12	0.21

**Table 2:** Adsorption kinetics parameters for adsorption of Cr(VI), Pb^2+^, and Cu^2+^ on ACNC.

Models	Parameters	Cr(VI)	Pb^2+^	Cu^2+^
Pseudo first-order	R^2^	0.660	0.614	0.598
	k_1_ (h^−1^)	0.466	0.388	0.415
	q_e cal_ (mg/g)	5.981	7.863	7.155
Pseudo second-order	R^2^	0.999	0.999	0.999
	k_2_ (g/mg·h)	0.122	0.162	0.112
	q_e cal_ (mg/g)	64.103	51.813	45.660
q_e exp_ (mg/g)		63.608 ± 1.645	51.571 ± 2.271	45.364 ± 2.072

**Table 3:** Calculated isotherms parameters for the adsorption.

Models	Parameters	Cr(VI)	Pb^2+^	Cu^2+^
Langmuir	R^2^	0.971	0.905	0.928
	K_L_ (L/g)	0.0434	0.0331	0.0299
	q_max_ (mg/g)	77.560	65.215	58.501
Freundlich	R^2^	0.809	0.709	0.759
	K_f_ (mg/g)	15.243	10.711	8.870
	1/n	0.296	0.321	0.333
Sips	R^2^	0.987	0.995	0.980
	q_s_ (mg/g)	70.503	54.115	49.600
	K_s_ (mL/mg)	0.0164	0.0127	0.0271
	m	1.378	2.119	1.799
	q_e exp_ (mg/g)	67.216 ± 1.688	53.258 ± 1.787	47.913 ± 1.267

**Table 4:** Comparison of the adsorption capacity of nanocellulose-based adsorbents with this work.

Adsorbents	Metal Ions	q_e_ (mg/g)	Reference
Amination CNCs with acrylamide and ethylenediamine	Cr(VI)	2.77	[33]
Polycysteine-grafted CNFs	Cr(VI)	87.5	[34]
carboxylic nanocellulose	Cu(II)	75.0	[35]
Pristine CNCs	Pb(II)	9.42	[9]
	Cu(II)	19.7	[8]
TEMPO-oxidized CNFs modified with PEI	Cu(II)	52.3	[36]
TEMPO-modified CNF	Cu(II)	23.8	[37]
Diethylenetriamine- BC (EABC)	Pb(II)	12	[38]
	Cu(II)	63.1	
ACNC	Cu(II)	49.6	This work
	Pb(II)	54.1	
	Cr(VI)	70.5	

## Data Availability

Data available upon request to corresponding author: xqh@qlu.edu.cn.

## Sample Availability

Samples of the extracts or compounds are available from the corresponding author: xqh@qlu.edu.cn.
